# Research Coproduction: How Can Coproduction Teams Increase Traffic on the Pathway to Impact? Comment on "Research Coproduction: An Underused Pathway to Impact"

**DOI:** 10.34172/ijhpm.8804

**Published:** 2024-11-18

**Authors:** Emily R. Ramage, Erin Bicknell, Saran Chamberlain, Brooke Parsons, Catherine M. Said, Elizabeth A. Lynch

**Affiliations:** ^1^The Florey Institute of Neuroscience and Mental Health, Heidelberg, VIC, Australia.; ^2^Department of Physiotherapy, The University of Melbourne, Parkville, VIC, Australia.; ^3^Physiotherapy, Western Health, St Albans, VIC, Australia.; ^4^Physiotherapy, Royal Melbourne Hospital, Melbourne, VIC, Australia.; ^5^Independent Researcher, Adelaide, SA, Australia.; ^6^College of Nursing and Health Science, Flinders University, Adelaide, SA, Australia.

**Keywords:** Coproduction, Codesign, Collaborative Research, Impact, Implementation Science, Knowledge Translation

## Abstract

The editorial by Rycroft-Malone and colleagues *Research Coproduction: An Underused Pathway to Impact*, explores the challenges and opportunities of coproduction to deliver research with impact. We, apply our experience as coproducers of research to present strategies that may accelerate uptake and increase traffic on the road to research impact. In doing so, we emphasise the importance of consistent terminology around coproduction, reporting impact metrics, diversity in research partnerships, and the careful consideration of researcher partners. Further, our commentary suggests practical strategies for teams to align their work with the principles of coproduction, and opportunities to support systems-level change to facilitate coproduction.

 Research impact is the effect research has on health, culture, environment, public policy or national security resulting from the translation of research.^1^ Barriers to translating evidence and impact remain despite positive progress in the field, resulting in research waste, and poorer health outcomes. The editorial by Rycroft-Malone and colleagues provides important insights for engaging the users of research, ie, “knowledge users” through coproduction to help close the gap between evidence and practice. The editorial emphasises the significant multilevel challenges facing coproduction research, these may be considered the metaphorical “potholes” deterring traffic on the road to impact. Addressing the challenges emphasised in the editorial could see great improvements in the relevance of research and its implementability. We aim to highlight potential actions coproduction teams may use to help tackle these barriers to coproduction at the micro, meso, and macro levels.

*At the micro level* Rycroft-Malone highlight that coproduction team members require the capacity and capability to engage in meaningful research coproduction. Specifically, the editorial identifies the importance of strong communication skills, emotional intelligence and interpersonal skills in coproduction. It is important that coproduction teams do not oversimplify the interpretation of this message and exclude specific cohorts of knowledge users without these skills. We contend that coproduction teams should ensure there are members with the skills to support genuine engagement and well-being of all members and adequate resources to optimise the partnership. For example, interpreters and translators can be used to support communication in research for ethnically diverse communities^2^ but increase both time and costs. Training team members in supported conversation techniques may facilitate inclusion of people with aphasia (a communication impairment).^3^ Strategies to support coproduction team members who lack high levels of emotional intelligence or interpersonal skills is lacking in the literature. Strategies to support relationship building with coproduction teams have been suggested, such as providing dedicated time and space for relationships to develop^4^; and, role definition and expectations.^5^ These methods may be most critical in teams where some members do not possess strong interpersonal skills or emotional intelligence. Furthermore, debriefing, clear processes for feedback and mechanisms for resolutions of disagreements or conflict which are actively utilised by the team may assist to identify issues within the team and allow them to be addressed early.^6^ Potential costs of coproduction can include burn-out, stress, and reputational damage^7^ it is foreseeable that by ensuring clear communication, and supporting interpersonal skills and emotional intelligence teams may minimise these costs of coproduction. In doing so, teams may maximise the uptake of coproduction and avoid breakdowns on the road to impact.

 The editorial highlights the potential for working with people with lived experience as patient partners to facilitate equity in research, and we strongly support this position. For effective partnership with people with lived experience, time is required to build trust and understanding. Mutual benefit is one of the aspirations of coproduction, and documented benefits of coproduction for people with lived experience include (but are not limited to) empowerment, skill acquisition, increased confidence, development of social networks and long-term professional, and personal relationships.^8^ However, it is important to understand that lived experience partners are unique in that their expertise of “lived experience” was not a choice, and partnering can come at an emotional or financial cost. Partnership for lived-experience members may bring up painful memories or traumatic events, and if remuneration is not provided can lead to loss of income or additional costs (eg, transportation to meetings or other research events). We advocate that coproduction teams with lived experience partners give careful consideration to what support is required for each individual and acknowledge the potential price of partnership to avoid tolls on the road to impact. We also recognise lived experience partners bring more than just their lived experience of a health condition, they also contribute their individual knowledge which may be professional, caring or life experience to enrich their contributions. We believe consideration of all expertise that end-users bring is needed when forming coproduction teams.

 Rycroft-Malone and colleagues advocate for broad consideration of partners for inclusion in coproduction research to optimise outcomes. Practically, to ensure knowledge users with the most relevant expertise are included, coproduction teams need to avoid partnerships based primarily on convenience, eg, who is eager and willing to form partnerships. Rather, teams should ensure all members bring relevant experience to the position. In addition to people with lived experience, engagement with clinicians, service providers and policy-makers should be considered. The road to impact is long, and these partners have power and capacity to implement and sustain research outcomes beyond the life of the project. However, competing priorities can make this challenging. Flexibility in the potential mechanisms for engagement from these groups may be required, for example providing alternative opportunities for team members to contribute to the project if they are unable to attend all meetings. Similarly, partnerships with community organisations are also critical but can take time to navigate, particularly in the early stages where team members may not have clarity on what support may be required or can be provided. Representation of community organisations and team membership may also change over time as personnel changes or people move roles. Therefore, specific strategies that support new team members to effectively partner part-way through the coproduction journey are needed.

 For diverse partnerships to thrive they must adhere to the road rules, that is, the principles of coproduction. Rycroft Malone and colleagues identify the principles of coproduction include “sharing power, valuing different sources of knowledge and viewpoints equally, reciprocity and mutuality, inclusivity, open communication, and attention to practical and financial considerations”^9^ (p. 2). These principles can conflict with traditional knowledge hierarchies of academy and therefore may require active “unlearning” by academic research partners. Teams may also need to consider specific strategies to minimise power differentials. For example, avoiding use of titles and mindful consideration of the ordering of team members names in documents. The need for shared power and equity, must also be balanced with the reality that all teams need leadership to ensure progress is made and outcomes achieved. While these features are not necessarily mutually exclusive, coproduction requires skilled leadership to ensure competing demands are met.

 The editorial by Rycroft-Malone and colleagues highlights that more research is needed to allow teams to understand the competencies, supports and training needed to optimise coproduction. Until such time as there is a robust body of coproduction methodology research, we advocate that emerging research can still provide important signposts along the road to direct coproduction approaches and preparation. For example, the Delphi technique has been used to identify competencies for integrated knowledge translation coproduction.^10^ Funding bodies and condition-specific foundations provide information and training in coproduction for researchers and people with lived experience.^11,12^ Furthermore, there is a growing body of published literature with exemplars, case examples or literature reviews that can provide practical guidance for coproduction teams. For example guidance exists for coproducing with indigenous peoples,^13^ coproducing interventions for clinical trial,^3^ influencing policy or community awareness,^14^ coproducing with immigrant healthcare users,^15^ or with graduate or early career researchers.^16^ We encourage coproduction teams to critically appraise current evidence that does exist in the field to enhance their own methods and processes.

*At a meso-level *the editorial calls for significant change within academy to realise the full potential of coproduction, noting that academy is currently “operating within a system that is largely counterproductive to meaningful research coproduction”^9^ (p. 2). While individual research teams may lack influence to make major shifts in the global academy, we believe they have potential to support incremental change that will help address barriers to future coproduction. By helping to break-down barriers to coproduction at an institutional level coproduction teams can facilitate a smoother and more efficient pathways to impact.

 The editorial suggests traditional research metrics can disincentive research coproduction, and therefore, may be the speed humps on the road to research impact. Academic institutions are moving to include impact as an indicator of success to capture the broader benefits of coproduction.^17^ We urge coproduction researchers to report the impact of their research to reinforce capacity of coproduction, and importantly, normalise reporting of impact as a key research metric. Practically, this may involve coproduction academics reporting website metrics of coproduced resources^18^ or media engagement and coverage.^14^

 Rycroft-Malone and colleagues suggest current academic and ethical review processes do not accommodate the realities of coproduction, which include iterative processes and partners without academic training. Coproduction researchers are positioned to lead change at their institutional level to introduce new or alternative systems to facilitate coproduction and therefore its uptake and impact. At a practical level, this may involve coproduction teams working and negotiating with their institutions and human research ethics review committees to develop local solutions to enhance the flow of coproduction traffic through research processes. However, this is not always straightforward and depending on the circumstances, may rely on strategic communication, education about the value and purpose of coproduction or even activism to create change. There are also practical steps teams could consider to minimise time burdens related to ethical approval process. For example, spending time early to plan the coproduction journey by iteratively and comprehensively defining the research questions and processes^3,18^ teams may reduce the need for ethical amendment.

 The editorial highlights that context is a key determinant of how coproduction is operationalised within a project. We suggest coproduction teams closely consider their opportunity to shape the context to optimise collaboration across community, healthcare and academic institutions. Such actions may help break down historical silos and hierarchies that inhibit coproduction and provide infrastructure to sustain future collaborations and uptake of on the road to impact. For example, advocating for research and the consumer positions embedded within healthcare organisations and colocation of research and healthcare may also facilitate academic-community-healthcare partnerships. Once formed these positions could be leveraged for future collaboration.

*At the macro level* the editorial emphasises a need for a shift in societal expectations of how research is developed, and how knowledge is valued. Importantly, the editorial identifies research coproduction should give all partners “an equal voice and role to play throughout the research”^9^ (p. 1). This classification aligns with definitions of “codesign”^19^ and “cocreation”^20^ however these definitions lack consistency internationally. While this could be considered simply an issue of semantics, confusion regarding terminology and therefore what coproduction “is,” is a barrier to its understanding, uptake and a roadblock to impact through coproduction. We call for an internationally agreed and adopted definition for research coproduction, codesign and cocreation to help progress the field.

 Rycroft-Malone and colleagues advocate that coproduction will enhance research impact through the development of usable, relevant and therefore translatable research. However, to optimally accelerate the uptake of this “underused pathway” researchers need stronger evidence the coproduction road leads to their intended destination, impact. Evaluation of coproduction research must confront issues of limited comparability between studies due to variations in scale, context and aims of the research, and the significant resources (time and finances) needed for longitudinal evaluation. Further, there remains a lack of agreement on how to objectively evaluate the quality of coproduction (eg, was this true partnership or did it veer towards “tokenism”), and authors have suggested reporting guidelines on how to measure coproduction (encapsulating coproduction processes, experiences of those involved and coproduction outputs) could further advance the field.^21^ We acknowledge that arguing for more research to support coproduction creates a focus on “academic knowledge.” This recommendation is not intended to undermine or dimmish other sources of knowledge (eg, lived experience or contextual knowledge). However, from the pragmatic position of coproduction researchers and knowledge users aiming to accelerate the uptake of coproduction, we contend this is best achieved by using the “language” (research evidence) of those we wish to influence, ie, researchers, academics, healthcare workers, policy-makers, and broader society. Further, evidence supporting coproduction will help coproduction teams argue for necessary funding, that is, the fuel to drive coproduction projects.

 When limited resources constrain coproduction, the challenge of ensuring genuine, impactful partnership is often more challenging than is generally recognised.^4^ Rycroft-Malone and colleagues’ editorial highlights changes neeeded to provide an environment that understands, accommodates and adequately resources coproduction. Such change has the potential to support knowledge translation and, we believe, ultimately better healthcare. The current roadmap to impact is incomplete. We hope this commentary can be a vehicle to start conversations about practical strategies to use co-production to increase traffic on the road toward better health outomes.

## Ethical issues

 Not applicable.

## Conflicts of interest

 Authors declare that they have no conflicts of interest.
